# Case report of the rare Peters’ anomaly complicated with Axenfeld-Rieger syndrome: A case report and brief review of the literature

**DOI:** 10.1097/MD.0000000000021213

**Published:** 2022-01-14

**Authors:** Yong Meng, Guohua Lu, Yang Xie, Xincheng Sun, Liqin Huang

**Affiliations:** a Department of Ophthalmology, Changzhou No. 3 People's Hospital, Changzhou, Jiangsu Province, China; b Department of Ophthalmology, Changzhou No. 2 People's Hospital, Changzhou, Jiangsu Province, China.

**Keywords:** anterior segment dysgenesis, Axenfeld-Rieger syndrome, Peters’ anomaly

## Abstract

**Introduction:**

Peters’ anomaly (PA) and Axenfeld-Rieger syndrome (ARS) are typical classifications of anterior segment dysgenesis (ASD) and ascribed to congenital eye diseases that encompass developmental defects in anterior segment structures. The aim of this study is to discuss the unusual association between PA and ARS and to determine the results of penetrating keratoplasty combined with extracapsular cataract extraction and anterior vitrectomy for this unusual ophthalmic phenotype.

**Patient concerns:**

A 72-year-old female was referred to Changzhou No. 2 People's Hospital for a progressive decrease in visual acuity in both eyes in the past few decades.

**Diagnoses:**

The patient was diagnosed with PA with cone-shaped polar cataracts in the left eye based on a series of ophthalmic examinations. ARS with retinal detachment was diagnosed in the right eye 2 years prior.

**Interventions:**

Penetrating keratoplasty combined with extracapsular cataract extraction and anterior vitrectomy were performed to manage PA with cataracts in the left eye.

**Outcomes:**

Her best corrected visual acuity did not improve significantly after the operation. Patients with ARS and PA should be treated cautiously because of fundus lesions.

**Conclusion:**

This study revealed that cases with PA accompanied by iridocorneal adhesions, or other ocular anomalies, need to be treated cautiously for a very low success rate. It is of reference value for the evaluation of treatment prognosis for this joint occurrence of ophthalmic phenotypes.

## 1 Introduction

Anterior segment dysgenesis (ASD) disorders may display a wide variety of clinical manifestations affecting the cornea, iris, ciliary body, anterior chamber and lens. During development of the anterior eye segment, cells that originate from the surface epithelium or the neuroepithelium need to interact with mesenchymal cells, which predominantly originate from the neural crest. Failures of proper interaction result in a number of developmental disorders such Peters’ anomaly (PA), Axenfeld-Rieger syndrome (ARS) or aniridia.^[1]^ PA is characterized by a central corneal opacity with defects in the corneal endothelium, Descemet's membrane, and posterior stroma; adhesion of the iris and cornea are also present. The condition is bilateral in 80% of cases, carries a high risk of early onset glaucoma, and often causes a low visual outcome. The inheritance has been described as autosomal dominant or autosomal recessive, but it has also been described as sporadic.^[2]^ This variance suggests multiple causative genes for PA. Disease severity has been classified into mild, moderate, and severe forms. Mild disease is characterized by the presence of a normal iris and lens. Central iridocorneal adhesions or other iris defects, such as atrophy or abnormal vasculature, are observed in moderate disease. There may be coexisting ocular abnormalities, such as sclerocornea, microphthalmos, aniridia, coloboma, and persistent hyperplastic primary vitreous (PHPV). ARS is a rare autosomal dominant inherited disorder with a prevalence of approximately 1 in 200,000 affecting the development of the eyes, teeth and abdomen.^[3]^ The syndrome is characterized by complete penetrance but variable expressivity. Ocular phenotypes are mainly characterized by ASD of the eye, such as iris hypoplasia (IH), a prominent Schwalbe line, variable degrees of iridolenticulocorneal adhesions, corectopia, polycoria, and corneal opacity. The wide variety of systemic features observed in ARS patients include facial dysmorphisms, dental anomalies and redundant periumbilical skin.^[3]^

In 1977, Awan KJ first described the rare joint occurrence of PA and ARS as Peters-Rieger's syndrome; complicated ocular and systemic anomalies were also mentioned.^[4]^ In the literature, only 3 cases with an anterior segment phenotype between the 2 eyes of a patient with coexisting PA and ARS and pituitary homeobox 2 (PITX2) or forkhead box C1 (FOXC1) mutations have been reported. In this manuscript, we report the case of a 72-year-old female with an unusual ophthalmic phenotype, with ARS in the right eye and PA with cone-shaped polar cataract in the left eye. Other associated ocular and systemic disorders that have not been previously mentioned in the literature were identified. This is the first reported case of an unusual ophthalmic phenotype of Peters-Rieger's syndrome in China. The aim of this study is to discuss the unusual association between PA and ARS and to determine the results of penetrating keratoplasty for this unusual ophthalmic phenotype. This study will be useful in elucidating the mechanisms of a wide range of ocular phenotypes, and it is of reference value for the evaluation of treatment prognosis for this joint occurrence of ophthalmic phenotypes.

## 2 Case presentation

**Figure 1:**
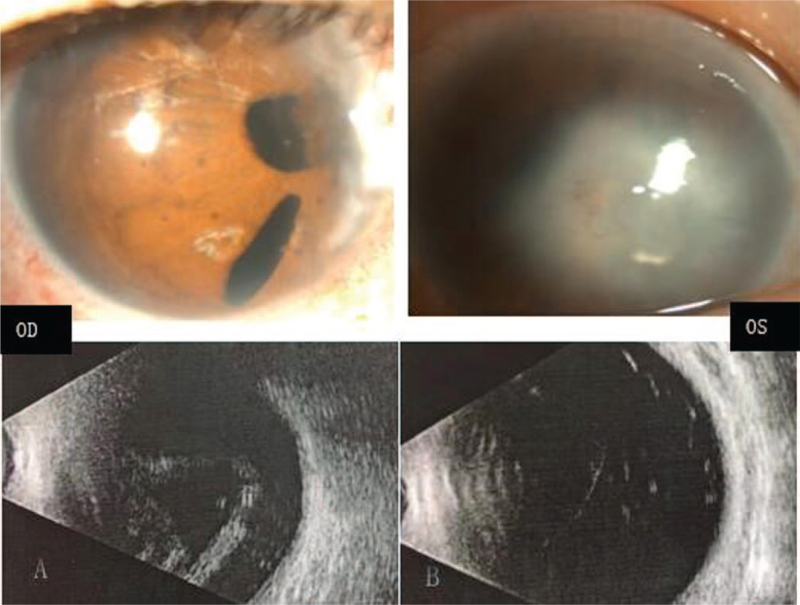
Ocular characteristics of the recruited patient. Biomicroscopic photograph of the anterior segment shows iris hypoplasia with polycoria and corectopia of her right eye (A upper). Examination of the left eye shows middle corneal opacification, central iridocorneal adhesions, and peripheral corneal vascularization (B upper). Type-B ultrasonic imaging of bilateral eyes: retinal detachment in the right eye and abnormal structure of the vitreous body in the left eye (A, B lower).

**Figure 2:**
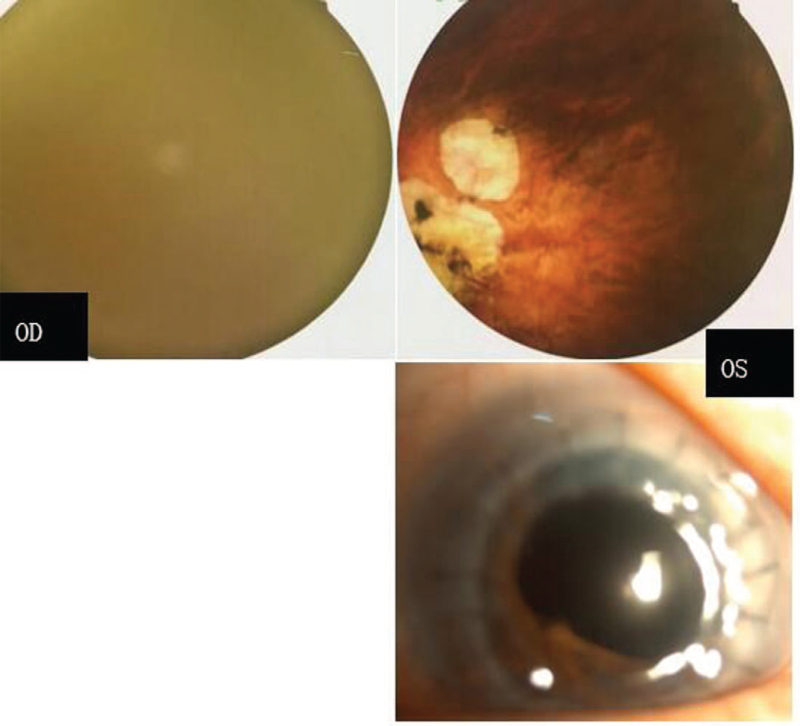
The posterior segment examination of the recruited patient after the operation: the right fundus photography is not clear because of nystagmus; the left eye shows hypoplasia of the optic nerve and hypoplasia of the macula half a year after the operation (upper). The transplanted cornea is transparent (lower).

The patient, a 72-year-old female, was referred to Changzhou No. 2 People's Hospital on December 7, 2018. She had a manifest horizontal nystagmus and exotropia, and the visual acuity of both eyes had been extremely poor over the past few decades. There were no familial cases, and she was diagnosed with ARS and retinal detachment in the right eye; ocular changes included IH and a prominent Schwalbe line, and iridocorneal adhesions at the angle of the anterior chamber were noted at another hospital 2 years prior. She underwent vitrectomy combined with phacoemulsification surgical for retinal detachment. Visual acuity showed significant improvement in her medical records; however, 1 year later, the patient experienced recurrent retinal detachment in the right eye. During this visit, her visual acuity was finger counting and light perception in the right and left eyes, respectively. The intraocular pressure measured with Goldmann tonometry was 14 mm Hg in the right eye and 17 mm Hg in the left eye. A-scanning demonstrated posterior staphyloma with axial lengths of 26.56 mm in the right eye and 27.73 mm in the left eye. Binocular diopters could not be detected because of nystagmus in both eyes. Examination of the right eye of the proband showed apparent evidence of IH, a prominent Schwalbe line, and iridocorneal adhesions in the angle of the anterior chamber. The abovementioned characteristics supported the diagnosis of ARS. Examination of the left eye showed severe ASD with middle corneal opacification, peripheral corneal vascularization and central iridocorneal adhesions. The central corneal opacity diameters were 7 mm×7 mm, and the fundus of the left eye could not be visualized. Examples of the anterior segment findings and type-B ultrasonic imaging are shown in Figure 1. Further systemic examination revealed characteristic midface anomalies, including telecanthus, broad, flat nasal bridge, a thin upper lip, and dental abnormalities. The patient and her family strongly demanded surgery for the left eye. The natural history and the surgery rates, risks and complications were explained in detail. Finally, the left eye was enucleated with penetrating keratoplasties, and anterior polar cataract with lens adhesions to the cornea was found during the operation. Therefore, penetrating keratoplasty combined with extracapsular cataract extraction and anterior vitrectomy was performed on the patient. The best corrected visual acuity (BCVA) was counting fingers 6 months after operation for the left eye; moreover, the transplanted cornea was transparent, and fundus examination showed evidence of foveal hypoplasia and hypoplasia of the optic nerve (Fig. 2).

## 3 Discussion

ASD has been classified into different subtypes, including ARS, PA, and aniridia. These conditions were thought to be caused by abnormal neural crest cell migration as it requires a successful combination of local factors, receptors, inductors, and signaling interactions between tissues such as the optic cup and periocular mesenchyme. ASD can be inherited either through autosomal recessive or autosomal dominant modes of inheritance. PA and ARS ophthalmopathy are rarely observed in both eyes of the same patient. In our study, the patient received vitreoretinal surgery 2 years ago in the right eye, and the postoperative BCVA showed significant improvement for horizontal nystagmus. Penetrating keratoplasty combined with extracapsular cataract extraction and anterior vitrectomy was performed for the left eye, and the BCVA was counting fingers with hypoplasia of the optic nerve and hypoplasia of the macula. PA has been classified into mild, moderate, and severe forms, with central iridocorneal adhesions or other iris defects coexisting with ocular abnormalities, such as sclerocornea, microphthalmos, aniridia, coloboma and PHPV, which are seen in severe disease. Severe disease has consistently been identified with an increased risk of graft failure for lenticulo-corneal adhesions. Yang et al reported that keratoplasty accompanied by lensectomy with anterior vitrectomy had an 87% failure rate in their series. In our case, the rare joint occurrence of PA and ARS occurred as a sporadic disorder. Eyes with severe disease, larger donor corneas, coexisting anterior synechiae and posterior segment anomalies have significantly poorer outcomes. The BCVA was counting fingers, although the transplanted cornea of the left eye was transparent after 6 months. Surgery should be undertaken with utmost caution, and posterior segment anomalies should be carefully considered before recommending corneal transplantation for PA with severe corneal conditions, coexisting anterior synechiae and nystagmus. Additionally, parents should be counseled extensively regarding the complications and guarded prognosis for success. In the present study, we first described the rare case of unusual phenotypic consequences for the coexisting ARS and PA in addition to a wide variety of extraocular disorders in China. This case is the first report of corneal graft survival after penetrating keratoplasty for this unusual ophthalmic phenotype. Thus, this study provides valuable insights regarding the outcome of corneal grafts in a patient with PA and ARS.

In this retrospective review, in 1977, Awan KJ first described the rare joint occurrence of PA and ARS as Peters-Rieger's syndrome. The associated ocular anomalies were dysversion of the optic disc, hypoplasia of the optic nerve, and downward ectopia of the macula.^[4]^ W Doward reported a patient with PA unilaterally with cataract and IH in the contralateral eye who had an A to T substitution at the invariant −2 site of the 3’ splice site of intron 3 of the PITX2 gene. An additional finding was mild left foveal hypoplasia. The patient had dental and umbilical abnormalities, while the intraocular pressure (IOP) was normal. Later, functional analysis demonstrated that these mutations were consistently associated with significantly PA and ARS in 1999.^[5,6]^ In 1990, Gerd E Holmstrom reported 3 families whose members had dominantly inherited anterior segment anomalies with phenotypic variability, including phenotype of the anterior segment with features of ARS in 1 eye but typical PA in the other eye.^[5]^ The IOP was within the normal range, and the putative pathogenicity of this anterior segment anomalies was later found to be caused by the mutation in exon 4 of PITX2 showing C ins 1083 by Rahat Perveen in 2000; in addition, dental and umbilical anomalies were present in this patient.^[6]^ Two of the abovementioned PITX2-affected patients had extraocular manifestations, including midface abnormalities with flattening of the midface and prominent lower lips. These phenotypic features are strong clinical indicators of PITX2 mutation in patients with ASD. The PITX2 gene is strongly associated with ARS associated with extraocular findings, and combined with the abovementioned cases, we speculated that these conclusions seem to apply to patients in the rare joint occurrence of PA and ARS, with screening of the PITX2 gene yielding negative results in the patient. PITX2 gene mutations can cause differences in development of binocular anterior segments in the same patient. One case with asymmetric anterior segment phenotypes between the 2 eyes of a patient with FOXC1 mutations has also been reported in the literature. In 2002, Honkanen RA first reported patient IV-1 with the joint occurrence of ARS and PA caused by a point mutation (Phe112Ser) in the FOXC1 gene. The patient had no systemic anomalies, the optic nerves were normal, and the IOP was normal; additionally, the patient never developed glaucoma.^[7]^ Berry et al demonstrated that FOXC1 and PITX2 physically interact, and this interaction may be essential for correct differentiation of neural crest cells during embryogenesis, thereby enabling normal anterior segment development.^[8]^ This idea is a potential explanation for why mutations in either gene can produce equivalent phenotypes. ARS and PA are posited to be allelic variants and part of the single disease spectrum of ASD rather than representing distinct entities. In our study, systemic manifestations were also present. However, screening of the PITX2 and FOXC1 genes in our patient was negative.

Neural crest cell disturbances also affect other parts of the body, which explains the ocular anomalies of the eye in the present patient. Of the 2 separate eye diseases, the patient exhibited wide phenotypic variation, and she was diagnosed with ARS, retinal detachment and exotropia in the right eye, while she was diagnosed with PA with cone-shaped polar cataract in the left eye. Unusual ocular anomalies have been reported with ARS or PA alone in the literature. Bhate et al and Park et al reported 2 cases with the presentation of hypoplasia of extraocular muscles associated with ARS^[9,10]^; extraocular muscles arise from the mesoderm, and impaired mesodermal development may explain these clinical manifestations. In 1989, Spallone et al reported 2 patients suffering from retinal detachment associated with proliferative vitreoretinopathy. In Kelberman's study, 1 patient had retinal detachment due to PHPV.^[11,12]^ For PITX2-positive ARS, a few reports indicated the presence of retinal detachment associated with proliferative vitreoretinopathy or PHPV.^[13–15]^ Akihisa Matsubara presented a hypothesis on the mechanism and critical period for the occurrence of PA, PHPV, and maldevelopment of the iris, which states that migratory disorders of neural crest cells from 4 to 7 weeks of gestation are responsible for the malformation complex. Three of the abovementioned patients with the rare joint occurrence of PA and ARS in this series had additional posterior segment anomalies, including foveal hypoplasia abnormalities, dysversion of the optic disc, hypoplasia of the optic nerve, and downward ectopia of the macula. These major ocular malformations suggest that the rare joint occurrence of PA and ARS is, at least in some cases, only one manifestation of a complex malformative syndrome affecting the developing eye as a whole rather than the anterior segment selectively. In our previous colleagues’ study, Xiuhua Jia and associates reported a novel mutation of paired box gene 6 (PAX6) in a Chinese 10-month-old male infant with new clinical features of PA. The infant had microcornea, microphthalmia, nystagmus, congenital corneal leukoma, iris dysplasia, and anterior polar cataracts in both eyes; ocular manifestations on B-scans revealed the abnormal structure of the vitreous body and optic nerve. The DNA of the patient had been screened for mutations in PITX2 and FOXC1, but none of the tests were positive.^[16]^ In 1977, Awan et al first described the rare joint occurrence of PA and ARS as Peters-Rieger's syndrome. The associated ocular anomalies were dysversion of the optic disc, hypoplasia of the optic nerve and downward ectopia of the macula, but they did not perform gene analysis for this family. In our study, the joint occurrence of PA and ARS was described. Other associated ocular disorders were exotropia, congenital nystagmus, congenital cataracts, and defects affecting the posterior segment, such as retinal detachment, foveal dysplasia and hypoplasia of the optic nerve. Screening of the PITX2 and FOXC1 genes was negative. In this retrospective review, the co-occurrence of cataracts with PA that has been reported in most affected cases clearly indicates that Pax6 is required for proper gene expression in the human lens. These cataracts evolve from small anterior or cataract lens opacities that are already present at birth. The wide expression of PAX6 in the developing eye encompasses the neurectoderm, surface ectoderm, and their derivatives and then continues in the adult retina, lens, and cornea. PAX6 is one of the master control genes in eye development. PAX6 functions as a transcription factor to regulate the expression of other genes during embryogenesis and in the adult eye and has been confirmed through the identification of a large number of mutations. Therefore, we propose that PAX6 is a key element that synchronizes the complex interaction of cell types of different origins, which are all needed for proper morphogenesis of the anterior and posterior eye, but screening of the PAX6 genes was negative in our patient. A number of genes are involved in migration and specification, such as PITX2, FOXC1, PAX6, C1P1B1, and PRDM5. The exact mechanism for the unusual association between PA and ARS is not clear. As we have outlined in this review, there could be some possible explanations as to why the severe ocular defects occur in this patient: first, there could be as-yet-undiscovered modifier mutations or in some other gene that affect the phenotype. Second, particular alleles of downstream target genes or cofactor genes might contribute to extensive ophthalmopathy in the patient, the association of PA with a variety of posterior segment phenotypes exhibiting much more severe ocular defects, as well as the overlap with several ARS, is further evidence that PA is a morphologic finding rather than a distinct entity. The aim of this study was to discuss the unusual association between PA and ARS, and it will be useful in elucidating the mechanisms of a wide range of ocular phenotypes and providing valuable insights about the outcome of operation in patients with PA and ARS. Hopefully, additional research in this area may eventually clarify the exact mechanism by which PA and ARS coincide in association with broad ocular disorder phenotypes. Future studies investigating the genetic interaction outlined in this review will help to further understand these complex diseases.

Nonetheless, there are some limitations in the present case:

1. Only 1 Peters-Rieger's syndrome patient was presented because it is an extremely rare eye disease in the ophthalmic clinic. We were unable to reach any convincing conclusions about the clinical features in the Chinese population. Thus, more subjects should be recruited in the future.
2. The patient had no family history, and no mutation could be found by gene screening. Further studies are now underway to determine whether other genes are responsible for the pathogenesis of the disease.
3. The major ophthalmologic concern in patients with PA or ARS is the risk of developing sight-threatening glaucoma, which occurs in approximately 50% of these patients. It is of interest that 3 of the abovementioned patients with joint occurrence of PA and ARS did not develop glaucoma. Therefore, based on the above evidence, it is reasonable to assume that patients diagnosed with PA and ARS may have multiple mechanisms that do not lead to glaucoma development. The reasons for this observation need to be further studied.

## Acknowledgments

We thank the patients and family members for their participation.

## Author contributions

**Data curation:** Xincheng Sun, Liqin Huang.

**Investigation:** Guohua Lu.

**Resources:** Yang Xie.

**Writing – original draft:** Liqin Huang.

**Writing – review & editing:** Yong Meng.
